# New insights into intestinal macrophages in necrotizing enterocolitis: the multi-functional role and promising therapeutic application

**DOI:** 10.3389/fimmu.2023.1261010

**Published:** 2023-09-28

**Authors:** Jiaqi Wei, Zhaoli Meng, Zhenyu Li, Dan Dang, Hui Wu

**Affiliations:** ^1^ Department of Neonatology, First Hospital of Jilin University, Changchun, China; ^2^ Department of Translational Medicine Research Institute, First Hospital of Jilin University, Changchun, China

**Keywords:** intestinal macrophage, necrotizing enterocolitis, preterm infant, inflammation, macrophage polarization

## Abstract

Necrotizing enterocolitis (NEC) is an inflammatory intestinal disease that profoundly affects preterm infants. Currently, the pathogenesis of NEC remains controversial, resulting in limited treatment strategies. The preterm infants are thought to be susceptible to gut inflammatory disorders because of their immature immune system. In early life, intestinal macrophages (IMφs), crucial components of innate immunity, demonstrate functional plasticity and diversity in intestinal development, resistance to pathogens, maintenance of the intestinal barrier, and regulation of gut microbiota. When the stimulations of environmental, dietary, and bacterial factors interrupt the homeostatic processes of IMφs, they will lead to intestinal disease, such as NEC. This review focuses on the IMφs related pathogenesis in NEC, discusses the multi-functional roles and relevant molecular mechanisms of IMφs in preterm infants, and explores promising therapeutic application for NEC.

## 1 Introduction

Necrotizing enterocolitis (NEC) is a severe gastrointestinal disease that affects over 90% of preterm infants (1). Globally, in neonatal intensive care units, the incidence of NEC is 7% in very low birth weight infants, with a fatality rate of 20–30% (2, 3). Given the unclear pathogenesis of NEC, it has hindered the further study of the disease. Therefore, there is a lack of specific and effective treatment methods, and current strategies are limited to supportive treatment. For infants showing continuous clinical deterioration, emergency surgery is required to remove the necrotic intestinal segments (4). Some surviving infants still face long-term sequelae (intestinal strictures, short bowel syndrome, and neurodevelopmental impairment) (5). Hence, there is an urgent need to explore the pathogenesis of NEC and search for new targeted therapies to enhance its management.

The pathogenesis of NEC has been extensively studied, and the current view is that multiple factors lead to the progression of the disease, including immature intestinal development, intestinal barrier dysfunction, gut microbiota dysbiosis, and excessive inflammation response (6, 7). The occurrence of NEC is regarded that intestinal mucosal bacterial colonization in preterm infants, which drives the inflammatory response to inappropriate innate immunity; abnormal immune regulation coupled with intestinal barrier damage triggers NEC. However, insufficient understanding of the complex interaction between the immature immune system and gut microbiota is one of the key limitations in NEC pathology to settle this devastating disease. Intestinal macrophages (IMφs), as an important part of intestinal innate immunity, are derived from recruitment and *in situ* differentiation of blood monocytes in the intestinal mucosa. A key pathological feature of human NEC is that many inflammatory cells, mainly Mφs, gather and infiltrate the intestinal mucosa (8). An acute drop in the peripheral blood monocyte count is also an early diagnostic basis for distinguishing NEC from other causes of feeding intolerance in neonates (9). An increasing number of studies have demonstrated that IMφs play a key role in maintaining dynamic intestinal balance, regulating inflammation, resisting pathogens, and eliminating aging or dead cells (10, 11). However, the beneficial or harmful effects of these cells on NEC remain unclear. As the main population of NEC patients, preterm infants have immature immune system, and their IMφs have unique phenotypes and functions that differ from full-term infants and adults. Therefore, IMφs in preterm infants may be involved in the occurrence of NEC in the mechanisms of maintaining intestinal barrier function, sensing changes in gut microbiota, and regulating intestinal immune response. Based on the features of IMφs in preterm infants, this review summarizes the role of IMφs in the pathogenesis of NEC and the molecular mechanisms involved in the development of the disease, to explore potential targets for the prevention and treatment of NEC.

## 2 Origin and functional features of IMφs in newborns

During early life, the newborn relies mainly on innate immunity as maturation of adaptive immunity lags behind that of innate immunity. Intestinal innate immunity consists of two key parts: the surface of the intestinal epithelium which acts as a physical barrier, and the immune cells, which respond quickly to potential threats (12–14). IMφs are the most abundant immune cells in the intestine and the first white blood cells to appear in the developing intestine. Mφs first appear in the fetal intestine at 11–12 weeks of gestation, increase rapidly during the 12-22 weeks period, and then continue to expand at a slower pace through early childhood (15). In the neonatal intestine, most of IMφs are constantly replenished by monocytes to recruit into intestinal lamina propria and transformed into lamina propria macrophages (LpMs) through cascade differentiation. Recruited monocytes express high levels of C-C motif chemokine receptor 2 (CCR2^hi^) and lymphocyte antigen 6 complex (LY6C^hi^), and low levels of CX3C chemokine receptor 1 (CX_3_CR1^low^). These monocytes first upregulate the expression of major histocompatibility complex class II (MHCII), then downregulate the expression of LY6C and CCR2, and finally upregulate the expression of CX_3_CR1 to differentiate into mature LpMs (16). During inflammation, monocytes differentiate into pro-inflammatory IMφs that lack up regulated CX_3_CR1 expression. The chemerin produced by epithelial cells is a chemoattractant that attracts Mφ precursors from the circulation into the intestinal mucosa of the fetus. Its expression in the small intestine is regulated by development; that is, it increases during the fetal period, peaks at 20-24 weeks, and then decreases to the initial low level at full term (15). The presence of chemerin may explain the initial development of IMφ populations. After birth, IL-8 and TGF-β secreted by epithelial cells and mast cells recruit blood monocytes to migrate to intestinal mucosa (17), which may be the reason for the constant expansion of the IMφ pool. LpMs exhibit high phagocytic activity, promote host defense and barrier integrity, and secrete IL-10 to expand FoxP3^+^ regulatory T cells (18, 19). A recent study indicated that there are a small number of self-renewal Mφ populations originating from embryonic precursors, and expressing the surface marker CX_3_CR1^hi^CD4^+^TIM4^+^ in the muscularis externa, that is muscularis macrophages (MMs), which have the ability to interact with the enteric nervous system (ENS) (20, 21). Because LpMs are the primary type of Mφs in the intestine of newborns, they are emphasized in the description in this review, unless otherwise stated.

During the neonatal period, the intestinal mucosa is often affected by environmental, nutritional, and gut microbiota exposure. Mφs in the lamina propria usually clear the bacteria that break through the intestinal epithelial barrier, because they are the first phagocytes in the innate immune system to interact with microbes and microbial products (17, 22). Because the phenotypic features and function of IMφs in fetuses or preterm infants are essentially different from those in full-term infants or adults, invasive bacteria cause abnormal immune responses in immature intestinal mucosa. First, neonatal Mφs show unique phenotypic features. Compared with adult mice (42 days), the Mφs of neonatal mice (<24 h) selectively lack the expression of F4/80, MHC II and costimulatory molecules (CD80 and CD86) (23), which corresponds to a decrease in the antigen presentation ability of Mφs in preterm infants. Second, IMφs of preterm infants exhibit a pro-inflammatory phenotype, because they quickly adapt to their functions by perceiving the surrounding microenvironment (24). Under the influence of TGF-β in the extracellular matrix of the lamina propria, fetal IMφs gradually acquire a non-inflammatory profile with increasing gestational age (25). Due to endogenous deficiency and a decrease in amniotic fluid and breast milk supplementation of TGF-β_2_ (26), IMφs in preterm infants show an immature, pro-inflammatory phenotype, that is, the release of various cytokines and inflammatory mediators (such as TNF-α, IL-1β, IL-6, IL-8, IL-10, and CXCL8) (8, 27, 28), while IMφs in full-term neonates and adults do not. Third, compared to full-term infants and adults, the phagocytic activity of IMφs in preterm infants is significantly lower, which leads to increased susceptibility to infection in preterm infants (29). In contrast, the enhanced ability of Mφs to generate reactive oxygen products in preterm infants may be a mechanism for overcoming immature phagocytosis (29). These functional features are in sharp contrast to the “inflammatory anergy” of Mφs in adult intestinal mucosa. Although adult IMφs retain avid phagocytic and bacteriocidal activity, these cells neither express innate response receptors (including CD14, CD89, CD64, CD32, CD16, and CD11b/CD18), nor produce pro-inflammatory cytokines (including IL-1, IL-6, IL-10, IL-12, TNF-α, or TGF-β) when exposed to bacterial products (22). Finally, compared to adult mice, the Mφs of neonatal mice exposed to Toll-like receptor (TLR) ligands showed a stronger chemotactic ability for phagocytes (monocytes and neutrophils) (30). The overresponse of neonatal Mφs may lead to the accumulation of other inflammatory cells and the nonspecific release of invasive substances stored by these cells, resulting in tissue injury related to the inflammatory state.

## 3 Role of IMφs in pathogenesis of NEC

**Figure 1:**
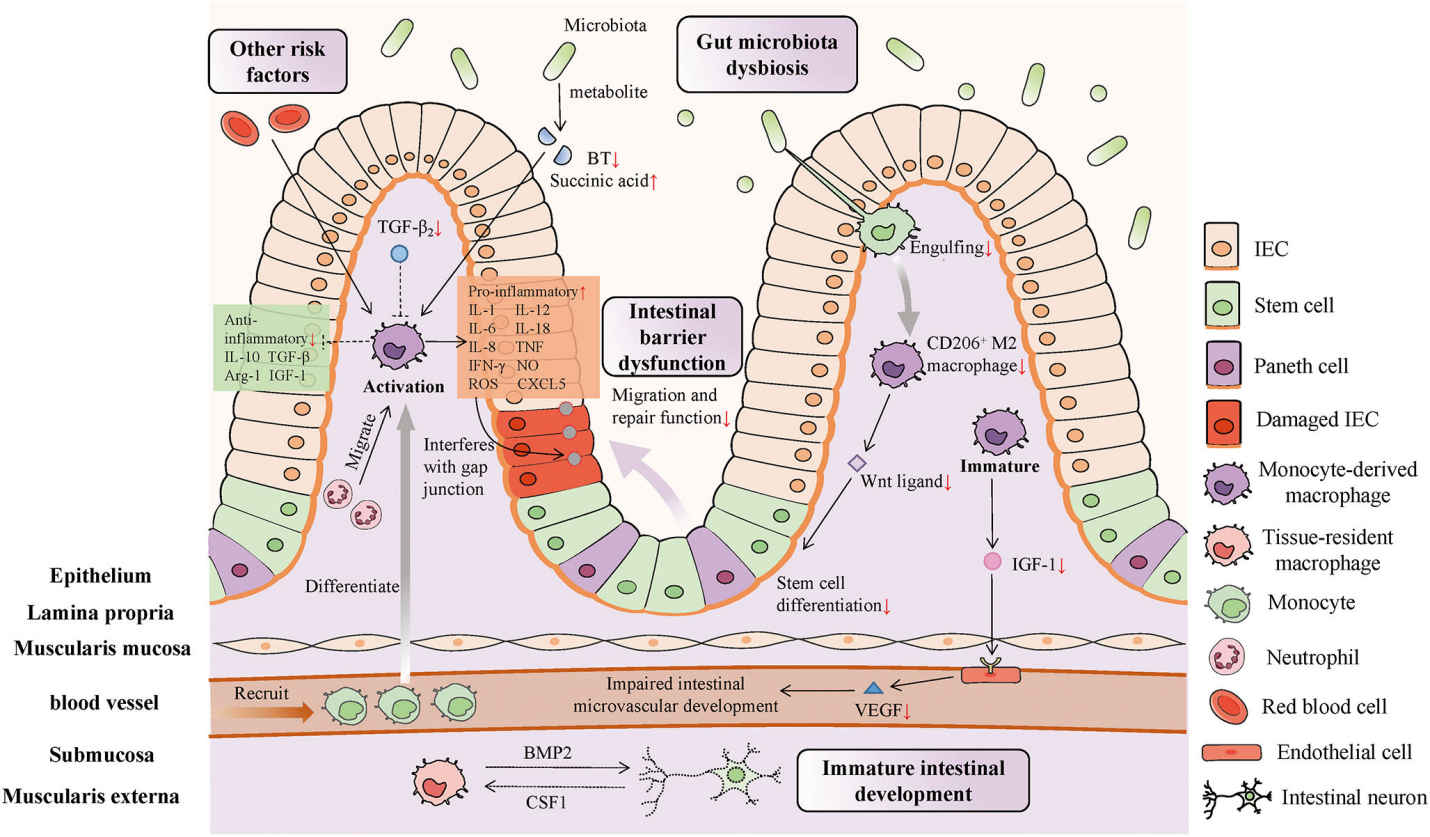
The multifunctional role and mechanism of IMφs in NEC pathogenesis. During intestinal inflammation of NEC, inflammatory monocytes and neutrophils are sequentially recruited to mount an immune response to the stimulations of environmental, dietary, and bacterial factors. (1) Immature intestinal development induces to the immature function of IMφs in preterm infants, including excessive inflammation response, immature intestinal motility and intestinal microvascular development. (2) The pro-inflammatory cytokines and regenerative signals released by IMφs affects the gap junction of IECs and repair function of intestinal epithelial barrier. (3) Alterations in gut microbiota makes the decreased engulfing of IMφs to them and reduced the differentiation of CD206^+^ M2 macrophage, leading to differentiation defect of stem cells. The metabolites of microbiota also regulate the inflammatory state of IMφs in NEC. (4) Red blood cell transfusion activates inflammation pathways in IMφs to expand inflammatory response of intestine.

The phenotype of IMφs correlates with intestinal development, and maintaining the homeostasis of IMφs is crucial for preserving the intestinal barrier and promoting favorable development of the gut microbiota. Disruption of this balance activates IMφs, and initiates the inflammatory cascade leading to NEC (**Figure 1**).

### 3.1 Relationship between IMφs and intestinal immaturity in NEC

The incidence and severity of NEC are strongly associated with preterm infants. Immature intestinal function, particularly immune defense, intestinal motility, and circulatory regulation, is a significant risk factor for NEC susceptibility in these infants (1). IMφs exhibit significant developmental plasticity and are instrumental in shaping neonatal intestinal immunity, motility, and circulation.

#### 3.1.1 Immune defense

The intestinal tract contains the largest number of Mφs in the body, that protect the host from detrimental microbes and contributes to maintaining intestinal homeostasis (31). In early life, IMφs play a crucial role in regulating intestinal immune defense (32), especially in eliminating bacteria from NEC. *Cronobacter sakazakii* (CS), previously known as *Enterobacter sakazakii*, is associated with clinical NEC cases resulting from formula milk contamination (33). Compared to wild-type mice infected with CS, Mφs-depleted mice infected with CS showed severe inflammation, villus destruction, and enhanced enterocyte apoptosis (34). Notably, the immune defense function of neonatal IMφs is regulated by developmental processes. An *in vitro* study demonstrated that IMφs of fetal mice express TNF-α upon lipopolysaccharide (LPS) stimulation, whereas neonatal and adult mice are tolerant to LPS (25). This is attributed to the gradual increase in TGF-β content in the microenvironment surrounding Mφs with pregnancy maturation, particularly the TGF-β_2_ isoform, which bolsters the inhibition of LPS-induced cytokine production in Mφs. Due to the weak host defense capability of Mφs in the immature intestine of preterm infants, “untimely” bacterial colonization of the intestinal mucosa may render these infants vulnerable to a strong inflammatory response, resulting in NEC. Consequently, enteral supplementation of TGF-β_2_ has been shown to prompt the differentiation of immature, highly pro-inflammatory Mφs into mature, non-inflammatory Mφs, thereby reducing the incidence of NEC (25).

#### 3.1.2 Intestinal motility

Preterm infants, either with or at risk of NEC, frequently exhibit intestinal dysmotility, which potentially increases the risk of intestinal injury (35). Recently, the regulatory role of MMs, a unique subtype of IMφs, in intestinal motility has attracted much attention (36). Intestinal motility is locally regulated by ENS and interstitial cells of Cajal (ICCs) (37, 38). The ENS comprises a network of neurons and enteric glia. In early life, the interactions between MMs and ENS begin to establish and influence each other’s maturation. The interactions reviewed in “MMs enhance ENS development through synaptic pruning and phagocytosis of abundant enteric neurons” (31), and “MMs and ENS regulate intestinal motility by exchanging signals via cytokines, growth factors, and neurotransmitters” (39). In adult mice, MMs regulate gastrointestinal motility by directly secreting bone morphogenetic protein 2 (BMP2) into intestinal neurons, which in turn produce colony stimulating factor 1 (CSF1), a growth factor essential for Mφ development, promoting the survival and differentiation of MMs (40). However, ENS is not the primary source of intestinal CSF1 until late in the preweaning period of mice, at the same point as MMs become the primary intestinal source of BMP2. Instead, CSF1 in the developing intestine primarily originates from endothelial cells and ICCs, while the maturation of intestinal neuro-immune regulation after birth may be influenced by environmental factors, such as intestinal microbes or diet (41). Therefore, the CSF1-BMP2 cross-communication in neonatal mice might be largely immature, which results in decreased intestinal motility. Importantly, restoring the gut microbiota during the early developmental stages could potentially reverse abnormal Mφ-neuron crosstalk, thereby mitigating the risk of intestinal disease (40). Currently, most researches focus on exploring the relationship between MMs and the pathogenesis of adult gastrointestinal diseases, such as postoperative ileus (42), and preterm infants in NEC with poor motility are similar pathophysiology to adult pathologies involving MMs (43). The developmental defects in the structure and function of ENS in preterm infants manifest as intestinal dysmotility (44), a proven pathogenic factor of NEC (45, 46). Therefore, the diminished ability of regulating intestinal motility of MMs to immature ENS may increase the risk of NEC in preterm infants, and improving the gut microbiota could potentially serve as a treatment to restore intestinal motility. Therefore, a deeper understanding of the interaction between MMs, ENS, and intestinal signaling could provide novel prevention and treatment strategies for NEC.

#### 3.1.3 Circulatory regulation

IMφs play an important role in embryonic angiogenesis and circulatory regulation (47, 48). IMφ-derived insulin-like growth factor-1 (IGF-1) is a signaling factor that promotes intestinal microvascular development in neonatal mice. When Mφs sense endothelial cells (EnCs) in juxtaposed villi, they produce more IGF-1, which promotes the expression of vascular endothelial growth factor (VEGF) and EnC proliferation, which is helpful for the development of intestinal microvascular (49). About the relation between intestinal microvascular development and NEC, it was reviewed in “Intestinal microvascular dysplasia in preterm infants significantly contributes to NEC, and defects in the intestinal VEGF-A/VEGFR2 signaling pathway reduce EnC proliferation and the density of the intestinal microvascular network” (50). Yan et al. found that IGF-1-producing Mφs are decreased in human NEC, and defective IGF-1 impairs intestinal microvascular development by reducing the expression of VEGF/VEGFR2, resulting in an increased susceptibility to NEC (49).

In addition, the immature intestinal circulation regulation in preterm infants is affected by perinatal hypoxia-ischemic injury, which changes the balance of microvascular tension and blood flow patterns in microvessels, causing intestinal ischemia and triggering the inflammatory cascade of NEC (51). Intestinal injury is mainly caused by restored oxygen levels and re-injection of blood flow. Intestinal ischemia-reperfusion (I/R) injury and its inflammatory response are mediated by innate immune components such as Mφs (48), and promoting the transformation of M1 macrophages to M2 macrophages may reduce intestinal I/R injury (52). Recently, it was found that *Lactobacillus murine* promotes the release of IL-10 from Mφs through TLR2, which can reduce I/R injury, suggesting that the gut microbiota are involved in the process of intestinal I/R injury (53).

### 3.2 Effect of IMφs on intestinal barrier function in NEC

The interaction between IMφs and intestinal epithelial cells (IEC) is essential for regulating homeostasis of intestinal barrier function (54). Human IECs produce IL-10 through Mφ-epithelial crosstalk mediated by TLR4 signaling in Mφs to maintain epithelial cell integrity (55). Destruction of the neonatal intestinal barrier is a key step in the occurrence and development of NEC (1), which shows not only a decline in the immature intestinal epithelial barrier structure to allow the invasion of pathogens, but also the lack of barrier repair ability after intestinal epithelial injury (56). Intestinal epithelial injury needs to be repaired by a series of events that coordinate IECs with intestinal resident and permeable Mφs (57). In the damaged intestinal mucosa, epithelial cells in the crypt near inflammation migrate collectively to the damaged site, while cytokines, growth factors, Wnt ligands, specialized pro-resolving mediators, and matrix metalloproteinases (MMPs) released by Mφs transmit regenerative signals to damaged IEC to promote epithelial cell proliferation and migration to restore the homeostasis and function of the epithelial barrier (58). Impaired Wnt/β-catenin pathway can affect IEC homeostasis and lead to intestinal regeneration dysfunction during NEC (59). Although TNF-α is generally considered a pro-inflammatory cytokine, recent evidence has shown that it promotes the repair of damaged mucosa by activating WNT/β-catenin signaling transduction, promoting IEC proliferation, and upregulating the expression of intestinal healing receptors (60). Nitric oxide (NO) is the earliest and most effective cytokine released by Mφs. In the inflammatory state of NEC, the sustained release of NO from the injured intestinal mucosa not only impairs gap junction communication between adjacent IEC mediated by connexin 43 (Cx43), but also inhibits IEC migration and mucosal repair by activating RhoA-GTPase (61, 62). IFN-γ also reduces gap junction communication by inhibiting the dephosphorylation and internalization of Cx43, thus inhibiting the migration ability of IEC and affecting intestinal healing during NEC (63). Improving our understanding of Mφ-IEC crosstalk is very promising for developing better strategies to treat NEC while maintaining barrier function.

In NEC, multiple types of regulatory cell death (RCD) of IEC lead to intestinal barrier dysfunction and induce local intestinal inflammation, including apoptosis, autophagy, pyroptosis, and ferroptosis (64–67). Several studies have shown that Mφs have a certain extent of association with the RCD occurrence of IEC in NEC. Interferon regulatory factor 5 (IRF5) is a master regulator for M1 macrophages and plays an important role in the induction of apoptosis (68, 69). Wei et al. found that IRF5 is upregulated in infiltrated macrophages in human neonates with NEC, and IRF5 deficiency in myeloid cells inhibits IEC apoptosis and prevents the destruction of the intestinal barrier in experimental NEC (70). In a rat model of NEC, heparin-binding epidermal growth factor (HB-EGF) inhibited M1 macrophage polarization and promoted M2 macrophage polarization, thereby reducing IEC apoptosis to protect the intestine barrier (71). Recent experimental data have shown that ferroptosis is an important way in which intestinal epithelial RCD appear in the pathogenesis of NEC. Bioinformatics analysis based on microarray data showed that ferroptosis in NEC is related to activated Mφs, and ACSL4, as a key regulator of ferroptosis, may regulate the immune function and inflammatory response of NEC by activating TLR (67).

### 3.3 Interaction between IMφs and gut microbiota in NEC

The interaction between immune cells and gut microbiota plays a crucial role in the maturation of the neonatal immune system (72). Gut microbial colonization drives CCR2-expressing monocytes to constantly replenish Mφs in intestinal mucosa, and the continuous sampling of intestinal microbes by Mφs helps to maintain intestinal immune balance (73). Gut microbiota dysbiosis occurs before NEC in preterm infants (74), alterations in gut microbiota can be perceived by antigen presenting cells, and Mφs directly interact with gut microbiota (75) and regulate Paneth cell differentiation (76). After birth, intestinal stem cells located at the base of the crypt begin to differentiate into enterocytes and secretory cells. Paneth cells are secretory cells, and their disruption is involved in the development of NEC (77). Recent studies have found that postnatal gut microbial exposure promotes Mφs to differentiate into M2-type non-inflammatory states and secrete the epithelial Wnt signaling required for the differentiation of stem cells into Paneth cells, thus maintaining mesenchymal niche cell (MNC) proliferation, a key part of the intestinal stem cell niche (78). However, antibiotic treatment in early life can lead to gut microbial disorders, affect Mφ differentiation and Wnt secretion, lead to proliferation defects of MNCs, and promote the pathogenesis of NEC, while Lactobacillus treatment or supplementation with exogenous CD206^+^ M2 macrophages can partially rescue Paneth cell differentiation deficiency and NEC-like phenotype (78). Maike et al. found that neonatal intestinal Mφ-derived S100A8-A9 can promote the co-development of host gut microbiota and mucosal immune system, while the loss of fecal S100A8-A9 is associated with diseases related to microbial disorders, such as NEC, and supplementation with these proteins may contribute to the development of preterm infants (79).

With changes in the gut microbiota of NEC, the metabolites change accordingly. The affected metabolites are usually regarded as a bridge between the microbiota and the host, which may regulate the function of IMφs through pattern recognition receptor (PRR) signaling and participate in the pathogenesis of NEC (80). Short-chain fatty acids are products of the bacterial fermentation of carbohydrates in the gut, which butyric acid (BT) induces a more effective immunomodulatory effect (81, 82). BT-treated mouse Mφs can down-regulate LPS-induced pro-inflammatory mediators by inhibiting the activity of histone deacetylase (83). BT has shown potential for early prediction and disease identification of NEC (84, 85), which is related to the immunomodulatory mechanism of BT in the immature intestine of preterm infants. BT inhibits IL-1β-induced inflammation in fetal intestinal organoids or fetal mouse intestines and significantly reduces the release of IL-8 or Mφ inflammatory protein 2 (86). Studies have shown that exogenous BT can alleviate intestinal pathological damage in NEC by inhibiting the expression of high-mobility group box 1 (HMGB1) and increase the proportion of beneficial intestinal bacteria (87). Therefore, the intestinal protective effect of BT on NEC may be achieved by exerting the anti-inflammatory effect of Mφs and inhibiting the downstream inflammatory pathway. Succinic acid is a metabolic signaling molecule that maintains intestinal homeostasis and immune regulation, and its specific surface receptor, succinate receptor 1 (SUCNR1), activates immune cells and participates in the response to intestinal inflammation (88). In the fecal samples of NEC children and NEC model mice, increased levels of succinate are related to changes in gut microbiota, and succinic acid activated Mφs by activating the SUCNR1-mediated HIF-1α signaling pathway, disrupting the balance of pro-inflammatory and anti-inflammatory mediators and leading to the progression of NEC (89).

### 3.4 Role of IMφs in other risk factors for NEC

The development of NEC also involves some other common clinical risk factors, such as severe anemia and blood transfusion exposure, and the exploration of its pathogenesis has confirmed the importance of IMφs. Evidence supports the association between RBC transfusion and adverse clinical outcomes, such as NEC, after anemia in preterm infants (90). However, there is no consensus on whether transfusion-related intestinal injury is caused by severe anemia or RBC transfusion (91, 92), which was recently investigated using a neonatal murine model of transfusion-associated NEC. Studies have found that severe anemia can independently promote the secretion of pro-inflammatory cytokines by Mφs, becoming a key initiation event that causes intestinal inflammation and barrier dysfunction (93). RBC transfusion activates these Mφs, and free hemoglobin, the RBC degradation product, mediates the production of ROS and inflammatory cytokines through TLR4 in Mφs and the NF-κB pathway downstream, resulting in a second blow to the intestinal tract (94). The occurrence of NEC requires continuous exposure to anemia and RBC transfusion, neither of which is an independent risk factor for intestinal injury (95). Selective depletion of inflammatory Mφs with diphtheria toxin or clodronate liposomes not only reversed the effect of anemia on intestinal barrier function, but also weakened the intestinal injury caused by RBC transfusion, and the inhibition of Mφ activation by anti-NF-κNPS also had the same effect (94). Early prevention and treatment of anemia and adoption of restrictive transfusion guidelines are key elements in the management of preterm infants, and intervention in the number and activity of Mφs may be a new target for the treatment of transfusion-related NEC. In addition, platelet transfusion increases the risk of NEC (96), and activated platelets may cause intestinal injury by releasing preformed vasoconstrictor factors and inflammatory mediators (97). Kopperuncholan et al. found that whatever medical NEC or surgical NEC in human both show increased the level of plasma tissue factor and the expression of tissue factor in IMφs. Neonatal IMφs release their unique tissue factor, which promotes platelet activation and aggregation by activating thrombin, and depleting platelets in microthrombosis formed in intestinal microvessels (98). Inhibition of TLR4 signaling in Mφs or targeted inhibition of thrombin by antithrombin nanoparticles can reverse NEC intestinal injury (98).

Therefore, IMφs act the multi-function role in NEC pathogenesis, and maintaining the homeostasis of IMφs may protect from NEC (**Table 1**).

**Table 1:** Role and therapeutic application of IMφs in NEC pathogenesis.

NEC pathogenesis	Normal role of IMφs	Impact of IMφs on NEC	Therapeutic application
Immature intestinal development			
Immune defense	Eliminate bacteria	Poor tolerance to bacteria of IMφs because of TGF-β_2_ deficiency	Enteral supplementation with TGF-β_2_
Intestinal motility	Regulate intestinal motility via the interaction between muscularis macrophages with ENS	Diminished ability of regulating intestinal motility of MMs to immature ENS	Improve gut microbiota
Circulatory regulation	Promote embryonic angiogenesis	Impaired intestinal microvascular development caused by defective IMφs-derived IGF-1	Exogenous supplementation with IGF-1
	Mediate intestinal I/R injury and its inflammatory response	Intestinal I/R injury caused by differentiation of M1 macrophages	Regulate gut microbiota
Intestinal barrier dysfunction			
	Maintain intestinal barrier function via interaction between IMφs and IECs	Impaired epithelial barrier repair signaling released by IMφs	Generate IMφ-IEC crosstalk
Gut microbiota dysbiosis			
	Perceive alterations in gut microbiota	Reduced M2 macrophage differentiation induced by gut microbiota dysbiosis, leading to differentiation defect of stem cells	Lactobacillus therapy or exogenous supplementation with CD206^+^ M2 macrophages
Other risk factors			
Severe anemia and RBC transfusion	–	Activated inflammation pathways in IMφs induced by RBC transfusion	Depletion of inflammatory IMφs Inhibition of macrophage activation
Platelet transfusion	–	Platelet activation and aggregation via tissue factor released by neonatal IMφs	Inhibition of TLR4 signaling in Mφs

## 4 IMφ-related molecular mechanisms during NEC development

### 4.1 Mφ polarization

Mφs could be polarized into different subtypes to play different biological functions under the action of different induction factors. Generally, two subtypes of polarized Mφs are classical macrophages (M1) and alternative macrophages (M2), at the same time the polarization is reversible (99). As the initiator of inflammation, M1 macrophages activate TLR4 and up-regulate NF-κB signaling when stimulated by LPS and release pro-inflammatory cytokines such as IL-1β, inducible nitric oxide synthase) and TNF-α to form an inflammatory microenvironment. In contrast, IL-4/IL-13 induce Mφs to polarize M2 macrophages and up-regulated the expression of IL-10, TGF-β, IGF, and other molecules to exert the functions of anti-inflammation and tissue repair (100). Preterm infants are vulnerable to a large amount of LPS, and the increased levels of IFN-γ and TNF-α in the intestines of children with NEC, that can provide sufficient stimulation for the intestinal mucosa and lamina propria Mφs, and provide a prerequisite for M1-type polarization.

Many studies have shown that M1 macrophages promote the development of NEC, and inhibiting M1 polarization and promoting M2 polarization reduces the incidence of NEC (70, 71, 101). HB-EGF prevents M1 and promotes M2 polarization by activators of transcription 3, thereby protecting the intestine from NEC (71). Recent studies have shown that IRF5 is a key regulator of neonatal M1 macrophages (102). Myeloid-specific deficiency of IRF5, which is associated with reduced M1 macrophage polarization and systematic inflammation, dramatically prevents experimental NEC (70). Glutaredoxin-1 deficiency promotes the inactivation of NF-κB, which attenuates the recruitment of monocytes and M1 macrophage polarization and protects against NEC-like intestinal injury (103). Hydrogen promotes the polarization of Mφs from M1 to M2 by inhibiting the expression of NF-κB p65 in the nucleus, thus reducing the severity of NEC (104). Therefore, polarization of Mφs may be a key factor in determining the regression or progression of NEC.

### 4.2 Mφ-related inflammatory signaling pathways

In the process of pathogen invading the intestinal mucosa, IMφs can recognise and bind pathogen-related molecular patterns and damage-related molecular patterns (DAMPs), because they express PRRs of cellular surface and intracellular, such as TLRs, nod-like receptors (NLRs), and C-type lectin receptors (10, 105). Therefore, pathogens and tissue destruction can be detected rapidly and efficiently. The repair and homeostatic functions of IMφs can be destroyed by persistent damage, resulting in a causal relationship between Mφs and the inflammatory state of disease (106).

**Figure 2:**
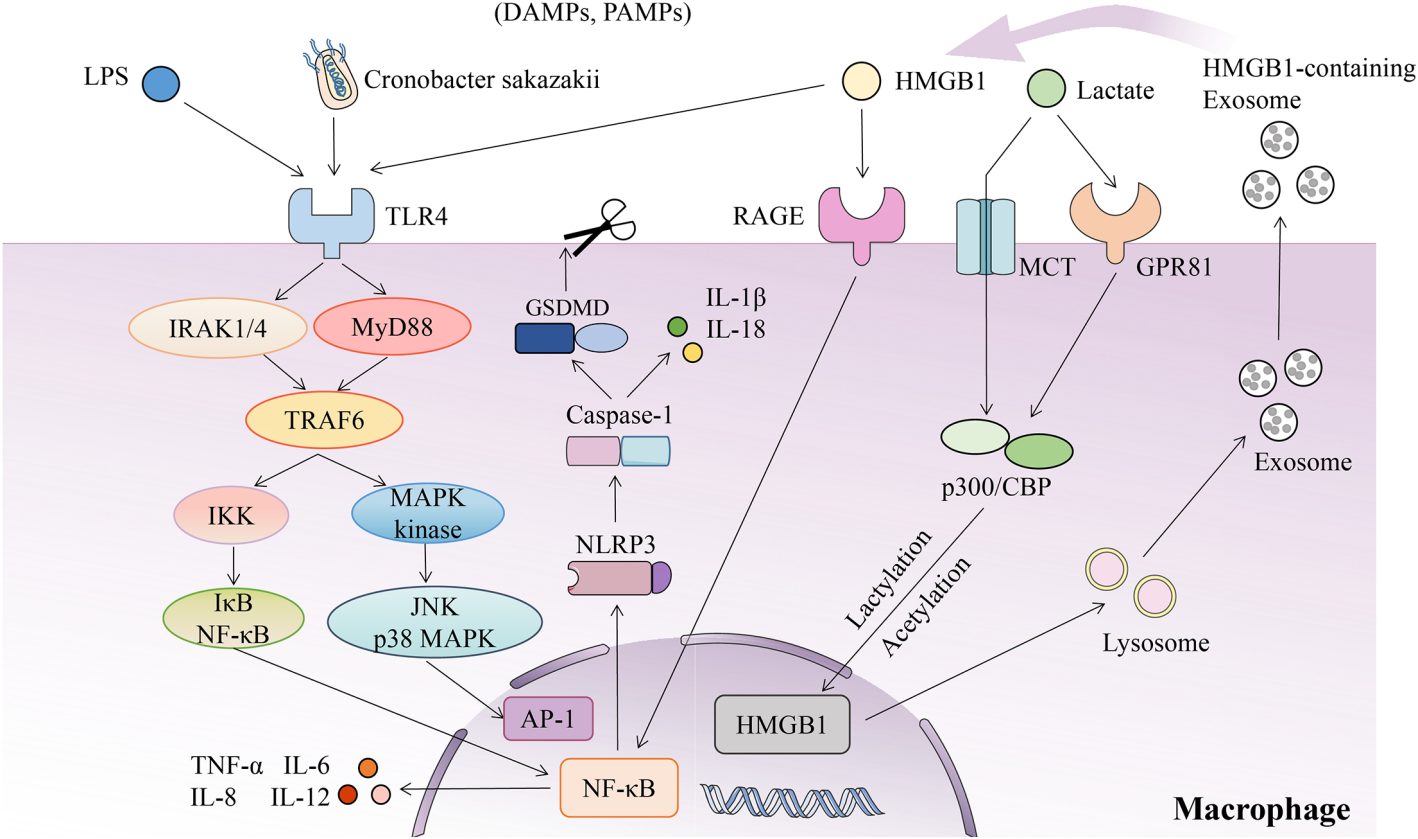
Mφ-related inflammatory signaling pathways in NEC. During NEC, Mφs are activated under the stimulation of DAMPs/PAMPs, such as LPS, Cronobacter sakazakii, HMGB1 and lactate, that initiates the intestinal inflammation leading to NEC. (1) In Mφs, LPS binds to TLR4, then recruits MyD88 and IRAK1/IRAK4. These molecules activate TRAF6 which in turn activates the IKK complex and MAPK kinase. MAPK kinase subsequently phosphorylates JNK and p38 MAPK, which activate the transcription factor AP-1. The IKK complex allows hosphorylation of IκB and subsequently degraded following ubiquitination, resulting in the activation of NF-κB and the release of pro-inflammatory cytokines. (2) CS induces TLR4/MyD88 signaling in Mφs and up-regulates the expression of NF-κB, which triggeres NEC through the NLRP3/caspase-1 pyroptosis pathway. (3) HMGB1 promotes intestinal inflammation in NEC by activating NLRP3 through TLR4 and NF-κB signaling pathways; and HMGB1 promotes chemotaxis through RAGE pathway and activates NF-κB signaling pathway to induce inflammation. (4) Mφs uptake extracellular lactate via MCT to promote HMGB1 lactylation via a p300/CBP-dependent mechanism. Extracellular lactate is also recruited to the nucleus through GPR81 to stimulate the acetylation of HMGB1. Lactylated/acetylated HMGB1 is released into extracellular through exosomes secreted by Mφs to continue as DAMP.

During NEC, Mφ-related inflammatory signaling pathways are involved in pathogenesis (**Figure 2**). The functional PRRs in Mφs includes TLRs and NLRs. The neonatal intestine is sensitive to LPS derived from gram-negative bacteria (107). In Mφs, LPS binds to CD14, which in turn binds to the TLR4/MD-2 complex, recruiting MyD88 and IRAK1/IRAK4. These molecules activate TRAF6 which in turn activates the IKK complex and MAPK kinase. MAPK kinase subsequently phosphorylates JNK and p38 MAPK, which activate the transcription factor AP-1. The IKK complex allows phosphorylation of IκB and subsequently degraded following ubiquitination, resulting in the activation of NF-κB and the release of pro-inflammatory cytokines (108). The importance of TLR4 signaling in IMφs has been confirmed in different mouse models of NEC. Inhibition of TLR4 signaling in Mφs can reverse the occurrence of NEC and NEC-related thrombocytopenia (98). In myeloid TLR4^-/-^ mice, after anemia-transfusion treatment, only newly recruited blood monocyte-derived Mφs were detected, but there was no intestinal injury (94). An MD2 inhibitor alleviated intestinal mucosal injury caused by inflammation in NEC by blocking TLR4-MD2/NF-κB signal axis (109). NLRP3 is an important member of the NLR-like receptor family. Under the stimulation of exogenous microbiota or endogenous danger signals, the activation of NLRP3 mediates the assembly of the inflammasome complex, triggering activation of caspase-1 and secretion of IL-1β and IL-18, and this process is called pyroptosis. Activation of NLRP3/caspase-1 pyroptosis pathway can destroy the intestinal barrier, guide neutrophils to the injured site, and promote the activation of Mφs, resulting in the recruitment of more inflammatory cells and the expansion of inflammatory response (110). In an *in vitro* experiment, CS induced TLR4/MyD88 signaling in Mφs and up-regulated the expression of NF-κB, which triggered NEC through the NLRP3/caspase-1 pyroptosis pathway (111). Melatonin and miR-146a-5p, which inhibit the activation NLRP3, are promising therapeutic targets in NEC (112, 113).

The function of Mφs is regulated by signaling pathways, such as NF-κB and MAPK activation (114, 115). NF-κB plays a central role in regulating the immune response of NEC (116, 117). Once TLR and other receptors are activated, IKKβ is activated, which leads to the nuclear translocation of NF-κB and the transcription of NF-κB target genes, including cytokines, chemokines, adhesion molecules, and cell surface receptors (118). In experimental NEC mice, the recruitment and differentiation of Ly6c^+^ monocytes into the intestine mediated by NF-κB signaling occurred within 24 h of induction, that is, the early stage of NEC. IKKβ is a key kinase mediating the activation of NF-κB. Pups with IKKβ deletion in Lysm^+^ cells prevented the NF-κB activation in monocytes and reduced NEC occurrence, whereas NEC was unabated in pups with IKKβ deletion in IECs (119). Krishnan et al. found that bacterial products specifically induced high Smad7 expression in IMφs of preterm infants, thus blocking normal autocrine induction of TGF-β_2_ in epithelial cells (120) and promoting NF-κB-mediated inflammatory signaling transduction by increasing the expression of IKKβ, ultimately activating inflammation (121). Another pathway involved in regulating Mφ activation in NEC is the MAPK signaling pathway, which is activated by pro-inflammatory cytokines and chemokines released by Mφs (122). Previous studies have found that the activation of p38 MAPK is differentially expressed and localized between normal neonatal intestinal segments and NEC intestinal segments (123) and that endotoxins cause enterocytes to release p38-dependent pro-inflammatory molecule COX-2, which potentiates the systemic inflammatory response during NEC (124).

The development of NEC is closely related to the cytokines released by activated Mφs, such as the pro-inflammatory factors TNF-a, IL-1, IL-12, IL-18, and CXCL5, and the anti-inflammatory factors IL-10 and TGF-β (27). In the process of NEC, intestinal injury is partly believed to be the result of TNF-α-induced activation of MMP pathway that can degrade the mucosal extracellular matrix (125). IL-1 binding to its receptor can trigger a series of signaling cascade reactions, leading to the activation of pro-inflammatory transcription factors, such as NF-κB and AP-1, which in turn induce the secretion of pro-inflammatory cytokines, such as IL-6, TNF, and IL-1 (126). The secretion of IFN-γ triggers the accumulation of IL-12 and IL-18 positive monocytes/Mφs, and these pro-inflammatory cytokines contribute a correlation with the progression of tissue injury in NEC (127). A significant decrease in the number of intestinal Mφs and mild NEC damage were detected in IL-18-deficient mice induced by NEC (128). Chemokine CXCL5, as the main initiator, recruited Mφ precursors from the circulation to the inflammatory intestinal mucosa, and this chemotaxis can be blocked by anti-CXCR2 (cognate receptor of CXCL5) antibodies (8). IL-10 inhibits intestinal inflammation because the specific knockout of IL-10R signaling in Mφs residing in the intestinal lamina propria causes severe spontaneous colitis (18). IL-10 also blocks the activation of NF-κB by inhibiting IκB kinase activity and NF-κB DNA binding activity to reduce intestinal mucosal inflammation (129). The inflammatory downregulation of TGF-β on Mφs inhibited the occurrence of intestinal mucosal inflammation in NEC (25).

HMGB1 is an important late inflammatory factor, which is actively secreted by activated Mφs or passively released from necrotic or damaged cells and plays a role as a DAMP (130, 131). Clinical data have confirmed that increased HMGB1 levels in fecal and serum assist in the early identification and prognosis of NEC (132, 133). The Mφ deactivator semapimod inhibited the secretion of HMGB1 and reduced the expression of its receptor for advanced glycation end products (RAGE), thereby reducing intestinal cell death and inflammatory response in a rat model of NEC (134). TLR and RAGE are important receptors for HMGB1, which itself not only affects NEC mucosal repair by inhibiting the migration of IECs in a TLR4-dependent manner (135), but also promotes intestinal inflammation in NEC by activating NLRP3 through TLR4 and NF-κB signaling pathways (136). In addition, HMGB1 promotes chemotaxis through RAGE pathway and activates NF-κB signaling pathway to induce inflammation; therefore, targeting HMGB1/RAGE/NF-κB pathway may be one of the measures to reduce intestinal inflammation (137). Lactate in circulating is the key regulator of HMGB1 secreted by Mφs, and the increase of neonatal lactate levels and the occurrence of sepsis are closely related to the mortality and prognosis of NEC (138, 139). During sepsis, Mφs uptake extracellular lactate via monocarboxylate transporters (MCT) to promote HMGB1 lactylation via a p300/CBP-dependent mechanism. Extracellular lactate is also recruited to the nucleus through G protein-coupled receptor 81 (GPR81) to stimulate the acetylation of HMGB1. Lactylated/acetylated HMGB1 is released into extracellular through exosomes secreted by Mφs to induce endothelial dysfunction (140). Specificity protein-1 was found to promote histone deacetylase 4-mediated deacetylation of HMGB1, thereby reducing intestinal barrier dysfunction, oxidative stress, and the inflammatory response induced by sepsis (141).

## 5 Therapeutic application of IMφs in NEC

By targeting the functional mechanism of IMφs in NEC, the following treatments may help improve the survival and prognosis of newborns. The first method involves changing the number or activity of Mφs. In a study using a neonatal murine model of transfusion-associated NEC, treatment with clodronate liposomes or anti-NF-κNPs to reduce the number or activity of Mφs, respectively, reversed the intestinal injury caused by anemia and RBC transfusion (94). Administration of semapimod, a Mφ deactivator, inhibited the expression of HMGB1 and RAGE in Mφs, thereby alleviating intestinal inflammation in NEC (134). The second method involves the regulation of the ratio of M1 and M2 macrophages. M1 macrophages increase LPS-induced human fetal small intestinal epithelial FHs-74 cell apoptosis, whereas addition of HB-EGF suppresses the pro-apoptotic effects and promote M1 to M2 macrophage polarization. Reducing M1 and increasing M2 polarization significantly protect the intestines from NEC (71). The third method involves anti-cytokine therapy. Anti-TNF antibodies can induce differentiation of M2 macrophages and participate in limiting inflammation (142). Blocking TNF-α signaling by using an anti-TNF-α monoclonal antibody (143), pentoxifylline (144), etanercept (145), or infliximab (146) significantly alleviated intestinal inflammation and tissue damage in NEC neonatal rats. Tocilizumab, a humanized monoclonal antibody against IL-6 receptors, inhibits intestinal inflammation in NEC by blocking IL-6-mediated signal transduction (147). The fourth method involves blocking specific signaling pathways activated by Mφs. In a mouse model of NEC, C34, an inhibitor of TLR4 signaling that tightly docks with the TLR4 co-receptor MD-2, suppressed TLR4 signaling in Mφs and reduced systemic inflammation (148). MCC950, which blocks NLRP3 inflammasome activation, ameliorates NEC-induced intestinal inflammatory injury and long-term cognitive impairment in mice (149). The fifth method involves feeding as much human milk as possible. Some bioactive components in human milk, such as lactoferrin, oligosaccharide, alpha-lactalbumin and glycomacropeptide, help Mφs to exert their intestinal immune function and significantly reduce the incidence of NEC. Monocytes isolated from umbilical cord blood of neonates treated with lactoferrin attenuate TLR4 signaling pathway, resulting in a diminished pro-inflammatory phenotype and decreased phagocytic activity of Mφs, which are involved in the protective mechanism of NEC (150). Human milk oligosaccharide activate M2 macrophages through exosomes as carriers to secrete cytokines, such as IL-10, IL-13, and IFA2, which can significantly reduce intestinal injury caused by bacteria in preterm infants (151, 152). Alpha-lactalbumin and glycomacropeptide also can inhibit the pro-inflammatory state of Mφs, suggesting that they may be related to the prevention of NEC (153, 154). The sixth method involves supplementation with probiotics and gut microbiota metabolites. Probiotics help restore the balance of the gut microbiota and play a critical role in the regulation of immune and inflammatory mechanisms (155). During early postnatal gut development, treatment with Lactobacillus induces the differentiation of M2 macrophages and secretion of Wnt ligands, thus maintaining the proliferation of MNC and reducing NEC severity (78). In addition, the effect of probiotics can be mediated by their metabolites, such as BT, which has anti-inflammatory effects and induces Mφ differentiation to prevent intestinal inflammation of NEC (81, 87).

## 6 Conclusion

IMφs play an important role throughout the development of NEC, including activation of inflammatory signals and release of cytokines to form a pro-inflammatory microenvironment in the early stage of NEC, recruitment of other inflammatory cells to expand inflammatory response in the progressive stage, and effects on IEC migration and barrier repair in the restoration stage. Previous studies have shown that the following methods are helpful in solving the problems of NEC therapy: changing the number and activity of Mφs, regulating the ratio of M1 and M2 macrophages, anti-cytokine therapy, blocking specific signaling pathways activated by Mφs, feeding human milk as much as possible, and regulating the gut microbiota. However, further research is required to fully understand these underlying mechanisms. In addition, the current obstacle in studying human intestinal immune development is that there are differences in intestinal development between humans and animal models, because mice are born with immature gastrointestinal mucosa (156). Therefore, understanding the phenotypic characteristics of IMφs and exploring the targeted changes in their functions are important steps in developing new strategies to improve the mucosal immunity of preterm infants with NEC.

## Author contributions

JW: Conceptualization, Writing – original draft, Writing – review & editing. ZM: Writing – original draft. ZL: Data curation, Writing – review & editing, Writing – original draft. DD: Writing – review & editing, Data curation. HW: Conceptualization, Writing – review & editing.
